# Counselling and psychotherapy service use in Chinese sexual minority populations: a nationwide survey

**DOI:** 10.1186/s12888-020-03010-3

**Published:** 2021-01-07

**Authors:** Yuanyuan Wang, Amanda Wilson, Zhishan Hu, Li Lu, Wengao Li, Ke Peng, Lijuan Wu, Ying Xin, Jack Drescher, Jianjun Ou, Runsen Chen

**Affiliations:** 1 National Clinical Research Center for Mental Disorders, Department of Psychiatry, and China National Technology Institute on Mental Disorders, The Second Xiangya Hospital of Central South University, Changsha, 410011 Hunan China; 2 Division of Psychology, Faculty of Health and Life Sciences, De Montfort University, Leicester, UK; 3 State Key Laboratory of Cognitive Neuroscience and Learning, Beijing Normal University, Beijing, China; 4 Team IETO, Bordeaux Population Health Research Center, UMR U1219, INSERM, Université de Bordeaux, Bordeaux, France; 5 Department of Psychiatry, Guangdong 999 Brain Hospital, Guangdong, China; 6 National Clinical Research Center for Cardiovascular Diseases, Shenzhen, Fuwai Hospital Chinese Academy of Medical Sciences, Shenzhen, China; 7 Department of Sociology, Peking University, Beijing, China; 8 Beijing LGBT Center, Beijing, China; 9 Department of Psychiatry, Columbia University, New York, NY USA; 10 Postdoctoral Program in Psychotherapy and Psychoanalysis, New York University, New York, USA

**Keywords:** Chinese, Sexual minority, Counselling, Psychotherapy, Service use, LGBT

## Abstract

**Background:**

This study investigated the prevalence and factors associated with counselling and psychotherapy service use among Chinese sexual minority populations.

**Methods:**

A nationwide cross-sectional study was performed using snowball sampling method, which led to the inclusion of 18,193 participants. Participants’ sociodemographic background, clinical, and psychological data were gathered. Multivariate logistic regression analysis was performed to explore any associated factors.

**Results:**

There were 2007 participants who had used counselling and psychotherapy service out of the total population. Among those who had used psychotherapy services, 80.2% participants perceived discrimination, 1.1% reported that they had been refused treatment by a counsellor and/or psychotherapist, 1.6% had experienced verbal harassment, and 8.4% reported that their counsellor and/or psychotherapist lacked knowledge and experience in treating sexual minorities. In addition, regression analyses indicated that those who were divorced/widowed, had religious beliefs, and those who had experienced discrimination, verbal harassment, and rejection for treatment by health professionals all had an increased likelihood of utilising counselling and psychotherapy service.

**Conclusions:**

Service providers and policy makers in China should improve the quality and availability of counselling and psychotherapy services to address the mental health needs of sexual minority populations.

**Supplementary Information:**

The online version contains supplementary material available at 10.1186/s12888-020-03010-3.

## Background

A national report roughly estimates that there are 75 million members of sexual minority communities (e.g. lesbian, gay, bisexual and transgender individuals, [LGBT]) in China [1]. Sexual minority populations are vulnerable to social prejudice, discrimination, violence, and lack of psychological support due to their sexual orientation, gender identity or expression [2, 3]. Compared with heterosexual and cisgender populations, sexual minority individuals experience higher rates of psychological distress, depressive and anxiety disorders, suicidality, and substance use disorders (i.e. binge drinking and tobacco smoking) [4]. Furthermore, sexual minority individuals are also more likely to report a lack of mental health support [5]. For example, compared to cisgender, heterosexual participants, transgender and bisexual individuals were 2.4 and 1.8 times more likely to report an unmet need for mental healthcare [6].

Several studies have investigated the prevalence and patterns of utilization of mental health service by sexual minority populations, however most of the studies have been conducted in western countries [7–9]. Compared to heterosexuals, sexual minorities, particularly lesbians and gay men, are more likely to consult mental health professionals and to report barriers to accessing services because of their sexual orientation [8, 9]. In terms of dissatisfaction with mental health services, LGBT groups show a significantly higher level of dissatisfaction than the heterosexual population group with 17.6% versus 8% respectively [10]. In both the general population and sexual minority communities, women are more likely to seek help from mental health services than men [6]. However, in regards to the quality of the service, around 21.7% of sexual minority women report being dissatisfied and 7.2% of women from the heterosexual population report being dissatisfied [10]. Additionally, the utilization of mental health services by sexual minority individuals’ may be further influenced by experiences of discrimination, low levels of social support, and a systemic hetero-normative healthcare environment [6].

To improve access to mental health care, reduce stigma, and manage physical and mental health comorbidities, China has invested substantially, and has made remarkable progress, in recent mental health reform [11]. However, counselling and psychotherapy services in China could greatly benefit from additional resources. There are currently less than 5000 qualified clinical counsellor/ and psychotherapists in mainland China, compared to more than 200,000 in the United States (US) [12]—a substantial difference, given that the total US population is only one quarter of China’s [12]. Compared with well-developed western countries, such as US, China has not established matured education, training, and job implementation procedures in the area of psychiatric service. In addition to having very limited access to counselling and psychotherapy services in China, individuals with psychological distress also have lower levels of self-awareness when it comes to understanding their own mental health status—and low levels of help-seeking—from mental health professionals. In response, the Chinese government and health authorities have made great efforts to alleviate related stigma and educate the public in the past two decades [13–15].

In terms of utilization of mental health services by Chinese sexual minorities, previous studies in Hong Kong indicate that LGBT individuals are more likely to seek help through informal channels, such as family members, friends, or other non-family LGBT community members when they need psychological support instead of a trained professional [16]. For those who have not disclosed their sexual identity to their families, friends and/or do not have a LGBT community network available nearby, social media (e.g. LGBT social networks) is as preferential platforms to express themselves because the environment is more safe, friendly, convenient, and ubiquitous [17].

Compared to heterosexuals, sexual minorities are at a higher risk of mental and physical health problems [18]. In China, very few studies are available exploring their experience with accessing physical and mental health services. In particular, little or no data exists about their help-seeking for counselling and psychotherapy services. Thus, this study aimed to investigate both the prevalence and factors associated with counselling and psychotherapy service utilization among Chinese sexual minority populations.

## Methods

### Participants and setting

This study was part of a large-scale epidemiologic survey on social attitudes towards LGBT individuals in China, which was undertaken from September to December 2015. Both face-to-face and an online survey were used for data collection. The initial recruitment processes occurred through LGBT community organizations, and the participants were approached by staff, with participants being obtained through convenience sampling. In total, 24 community organizations working with sexual and gender minorities, and several social media channels (such as, LGBT social networks, Weibo and Wechat) were involved during the recruitment of participants. No clinics were approached in an attempt to avoid anyone who may currently be in treatment for a serious psychiatric disorder. Across the 24 different community organizations, the staff in the LGBT community organizations distributed the questionnaire by providing an online link or they provided a hard copy of the questionnaire to the participants. After the initial recruitment, a snowball sampling method was used to recruit additional participants. Although multiple sampling strategies were applied (convenience, snowball, and respondent-driven sampling), the majority of participants were recruited through the online advertisement.

Each participant could only answer the questionnaire in person or online once to avoid duplicate responses from the same individual. The current study (secondary analysis) received approval by the Human Research and Ethics Committee of Second Xiangya Hospital, Central South University in China. The informed consent was provided in written format before participating in the survey. For online data collection, participants were required to confirm that they had read the information sheet, had time to ask questions and provide informed consent before being directed to the survey questions. Participants were ensured that their participation was completely voluntary and that their information would be kept confidential.

### Measures

Sociodemographic background (9 items), clinical items (2 items), and psychological (6 items) data were gathered from the participants. The researchers collected sociodemographic information such as, age, residency, marital status, educational background, employment status, and yearly income level using multiple choice questions. Participant’s HIV status and physical disability were assessed using binary questions. We also assessed participant’s psychological status by using six questions: 1) ‘Have you ever experienced psychological distress because of your sexual or gender minority status?’, 2) ‘Have you ever disclosed your sexual or gender minority identity to others?’, 3) ‘Have you ever experienced discrimination because of your sexual or gender minority status?’, ‘Have you ever been 4) refused treatment, 5) verbally attacked, or 6) sexually harassed by health professionals because of your sexual or gender minority status?’ These questions were assessed using a binary format of yes or no.

Participant’s counselling and psychotherapy experiences were evaluated by binary-response questions. All the questions used in the current study were based on previous findings and designed using expert consultation from the Beijing LGBT Center [19, 20]. First, participants were asked whether they had any previous counselling and psychotherapy experience. Those who answered yes were further assessed by five questions: ‘Have you ever experienced 1) verbal harassment, 2) sexual harassment, 3) any kind of discrimination from a counsellor and/or psychotherapist?’ 4) ‘Whether a counsellor and/or psychotherapist had refused to treat you?’, and 5) ‘Whether your counsellor and/or psychotherapist had sufficient knowledge or experience treating sexual minority populations?’

### Statistical analysis

Data was analysed using the Statistical Package for the Social Sciences version 16 (SPSS Inc., Chicago, Illinois). Normal distribution assumption was examined. First a comparison was performed using chi-square tests between the counselling and psychotherapy service use and non-use groups in terms of sociodemographic, clinical and psychological variables. Second, bivariate and multivariate logistic regression analysis with the ‘Enter’ method was used to calculate the crude and adjusted odds ratios (OR) and related 95% confidential interval (CI). In the multivariate analysis, service utilization was the dependent variable, while variables that significantly differed between the 2 groups in the univariate analyses were entered as independent variables. Multicollinearity was tested using Spearman’s Rho correlations. For all the statistical tests, the threshold of significance was set at 0.05 (two-tailed).

## Results

### Sample characteristics

Participants were recruited from 31 provinces in mainland China. In total 18,193 participants provided valid responses and were therefore included in the analysis. Specifically, 2066 (11.4%) participants identified as lesbian, 9491 (52.2%) as gay men, 3441 (18.9%) as bisexual, and 3195 (17.6%) as transgender. The mean age of the sample was 22.87 years (SD = 5.52). The majority of the participants were Han ethnicity, with 92.5% lesbian, 92.9% gay, 92.7% bisexual, and 92.2% transgender. Of the 18,193 participants included in the study, most were single (93.6%), living in urban areas (78.7%), well-educated (college/undergraduate and above: 73.2%), were not religious (82.6%), and were currently employed (57.2%). About 85.6% reported a negative HIV test result and 99.1% did not have a physical disability (see Table 1). The HIV status was collected as previous studies indicate that sexual minorities are at a higher risk of HIV infection [21]. However, in this study no significant differences were found for participants based on HIV or Disability status.

**Table 1:** univariate analysis of study variables in sexual minority population (*N* = 18,193)

Variables		Counselling/psychotherapy Service Use				
	Total N = 18,193	Yes *N* = 2007 (11.0%)	No *N* = 16,86 (89.0%)	X^**2**^	df	*P*-value
**Sociodemographic**						
**Population**						
Lesbian	2066 (11.4)	257 (12.4)	1809 (87.6)			
Gay	9491 (52.2)	1051 (11.1)	8440 (88.9)			
Bisexual	3441 (18.9)	356 (10.3)	3085 (89.7)			
Transgender	3195 (17.6)	343 (10.7)	2852 (89.3)	6.124	3	0.106
**Age (Years)**						
20 and below	7051 (38.8)	680 (9.6)	6371 (90.4)			
21–29	9274 (51.0)	1072 (11.6)	8202 (88.4)			
30 and above	1868 (10.3)	255 (13.7)	1613 (86.3)	29.521	2	**< 0.001**
**Residency**						
Urban	14,326 (78.7)	1686 (11.8)	12,640 (88.2)			
County	3026 (16.6)	254 (8.4)	2772 (91.6)			
Rural	841 (4.6)	67 (8.0)	774 (92.0)	37.432	2	**< 0.001**
**Marital Status**						
Married	879 (4.8)	99 (11.3)	780 (88.7)			
Single	17,022 (93.6)	1858 (10.9)	15,164 (89.1)			
Divorced/widowed	292 (1.6)	50 (17.1)	242 (82.9)	11.232	2	**0.003**
**Child Rearing**						
No	17,449 (95.9)	1927 (11.0)	15,522 (89.0)			
Yes	744 (4.1)	80 (10.8)	664 (89.2)	0.062	1	0.804
**Education**						
Primary or below	48 (0.3)	3 (6.3)	45 (93.8)			
Middle/High school	4840 (26.6)	384 (7.9)	4456 (92.1)			
College/undergraduate	12,040 (66.2)	1417 (11.8)	10,623 (88.2)			
Postgraduate	1265 (7.0)	203 (16.0)	1062 (84.0)	87.537	3	**< 0.001**
**Employment**						
No	10,398 (57.2)	1168 (11.2)	9230 (88.8)			
Yes	7795 (42.8)	839 (10.8)	6956 (89.2)	1.001	1	0.317
**Yearly Income (RMB**^a^**)** ^b^						
Less than 25,000	3516 (35.5)	367 (10.4)	3149 (89.6)			
25,001-49,999	2839 (28.7)	314 (11.1)	2525 (88.9)			
50,000-99,999	2391 (24.2)	258 (10.8)	2133 (89.2)			
More than 100,000	1145 (11.6)	179 (15.6)	966 (84.4)	24.832	3	**< 0.001**
**Religious Beliefs**						
No	15,036 (82.6)	1578 (10.5)	13,458 (89.5)			
Yes	3157 (17.4)	429 (13.6)	2728 (86.4)	25.449	1	**< 0.001**
**Clinical**						
**HIV Positive**						
No	15,581 (85.6)	1707 (11.0)	13,874 (89.0)			
Yes	325 (1.8)	49 (15.1)	276 (84.9)			
Unknown	2287 (12.6)	251 (11.0)	2036 (89.0)	5.518	2	0.063
**Physical Disability**						
No	18,022 (99.1)	1990 (11.0)	16,032 (89.0)			
Yes	171 (0.9)	17 (9.9)	154 (90.1)	0.209	1	0.648
**Psychological**						
**Psychological Distress**						
No	5228 (28.7)	405 (7.7)	4823 (92.3)			
Yes	12,965 (71.3)	1602 (12.4)	11,363 (87.6)	80.659	1	**< 0.001**
**Disclosure to others**						
No	8921 (49.0)	673 (7.5)	8248 (92.5)			
Yes	9272 (51.0)	1334 (14.4)	7938 (85.6)	216.945	1	**< 0.001**
**Refused by HP**						
No	18,077 (99.4)	1979 (10.9)	16,098 (89.1)			
Yes	116 (0.6)	28 (24.1)	88 (75.9)	20.432	1	**< 0.001**
**Verbal harassment by HP**						
No	18,031 (99.1)	1969 (10.9)	16,062 (89.1)			
Yes	162 (0.9)	38 (23.5)	124 (76.5)	25.711	1	**< 0.001**
**Sexual harassment by HP**						
No	18,126 (99.6)	1989 (11.0)	16,137 (89.0)			
Yes	67 (0.4)	18 (26.9)	49 (73.1)	17.178	1	**< 0.001**
**Discrimination Experience**						
No	3219 (17.7)	224 (7.0)	2995 (93.0)			
Yes	14,974 (82.3)	1783 (11.9)	13,191 (88.1)	66.107	1	**< 0.001**

*LGBT* lesbian, gay, bisexual, and transgender; *No* Number; *HIV* human immunodeficiency viruses; *HP* Health Professionals; ^a^US$1 = RMB7.03; ^b^: only in employed, valid *N* = 9891

There were 2007 (11.0, 95%CI: 10.6–11.5%) participants had used counselling and psychotherapy service. Among those who had used services, 1610 (80.2, 95%CI: 78.5–82.0%) participants had perceived discrimination due to their minority identity, 23 (1.1, 95%CI: 0.7–1.6%) reported that they had been refused treatment by a counsellor and/or psychotherapist, 33 (1.6, 95%CI: 1.1–2.2%) had experienced verbal harassment, and 12 (0.6, 95%CI: 0.3–0.9%) had experienced sexual harassment in counselling and psychotherapy. Meanwhile, 168 (8.4, 95%CI: 7.2–9.6%) participants reported that their counsellor and/or psychotherapist lacked sufficient knowledge and/or experience in treating sexual minorities. Additionally, among the 12,965 individuals (71.3, 95%CI: 70.6–71.9%) who reported psychological distress, 1602 (12.4, 95%CI: 11.8–12.9%) of them sought help from counselling and psychotherapy.

Results indicated that those who were older, living in an urban area, divorced/widowed, who had higher educational background, higher income levels, and those who had religious beliefs were more likely to seek counselling and psychotherapy. Those who had experienced psychological distress or discrimination, who disclosed their sexual minority identity to others, those who had ever been refused treatment, or verbally attacked or sexually harassed by health professionals were more likely to seek help from psychological professionals (see Table 1).

Using the results from the above Chi-Square tests, the 12 variables (6 sociodemographic and 6 psychological factors) that had a significant correlation with counselling and psychotherapy service use were introduced into the logistic regression as independent variables. These independent variables exhibited no significant multicollinearity (for all analyses the Spearman’s Rho correlation coefficient was < 0.4, see Table 2). All factors except for education level, showed significant association with service use in the bivariate logistic regression analysis (see Table 3 and supplementary materials).

**Table 2:** correlation between included variables

Correlations												
	1	2	3	4	5	6	7	8	9	10	11	12
1.Age	1	−.053^a^	−.197^a^	.267^a^	.329^a^	−.067^a^	−.005	−.040^a^	.032^a^	.024^a^	.010	−.248^a^
2.Residency		1	−.061^a^	−.241^a^	−.143^a^	−.046^a^	−.014	−.074^a^	.012	.002	.022^a^	−.029^a^
3.Marital Status			1	.009	−.079^a^	.014	.006	.071^a^	−.025^a^	−.011	−.007	.089^a^
4.Education				1	.280^a^	.053^a^	−.050^a^	.017^b^	−.023^a^	−.015^b^	−.016^b^	.024^a^
5.Yearly Income (RMB) ^a^					1	.009	−.025^b^	.013	−.028^a^	−.012	−.002	−.036^a^
6.Religious Beliefs						1	.017^b^	−.009	−.022^a^	−.020^a^	−.027^a^	.025^a^
7.Psychological distress							1	.046^a^	.001	−.023^a^	−.005	−.112^a^
8.Disclosure to others								1	.008	.019^a^	.011	.114^a^
9.Refused by HP									1	.161^a^	.109^a^	.037^a^
10.Verbal harassment by HP										1	.139^a^	.044^a^
11.Sexual harassment by HP											1	.028^a^
12.Discrimination Experience												1

^a^ Correlation is significant at the 0.01 level (2-tailed). ^b^. Correlation is significant at the 0.05 level (2-tailed). a: only in employed, valid N = 9891; HP: Health professional

**Table 3:** logistic regression of study variables in sexual minority population (*N* = 18,193)

	Counselling/psychotherapy service use					
Variables	Crude OR	95% CI	*P*-value	Adjusted OR	95% CI	*P*-value
**Sociodemographic**						
**Age (Years)**						
20 and below	Reference	–	–	–	–	–
21–29	1.225	1.106–1.355	**< 0.001**	0.977	0.792–1.204	0.825
30 and above	1.481	1.270–1.728	**< 0.001**	1.105	0.850–1.436	0.456
**Residency**						
Urban	Reference	–	–	–	–	–
County	0.687	0.598–0.789	**< 0.001**	0.887	0.737–1.067	0.202
Rural	0.649	0.503–0.837	**0.001**	0.885	0.635–1.234	0.471
**Marital Status**						
Married	Reference	–	–	–	–	–
Single	0.965	0.779–1.196	0.747	0.903	0.700–1.166	0.435
Divorced/widowed	1.628	1.125–2.355	**0.010**	1.551	1.051–2.289	**0.027**
**Education**						
Primary or below	Reference	–	–	–	–	–
Middle/High school	1.293	0.400–4.179	0.668			
College/undergraduate	2.001	0.621–6.447	0.245			
Postgraduate	2.867	0.883–9.315	0.080			
**Yearly Income (**RMB^a^**)** ^b^						
Less than 25,000	Reference	–	–	–	–	–
25,001-49,999	1.067	0.910–1.252	0.425	1.025	0.868–1.211	0.768
50,000-99,999	1.038	0.877–1.228	0.666	0.893	0.746–1.069	0.217
More than 100,000	1.590	1.311–1.928	**< 0.001**	1.221	0.987–1.512	0.066
**Religious Beliefs (ref: no)**	1.341	1.196–1.504	**< 0.001**	1.362	1.176–1.577	**< 0.001**
**Psychological**						
**Psychological Distress (ref: no)**	1.679	1.498–1.882	**< 0.001**	1.842	1.571–2.159	**< 0.001**
**Disclosure to others (ref: no)**	2.060	1.868–2.271	**< 0.001**	2.078	1.815–2.379	**< 0.001**
**Refused by HP (ref: no)**	2.588	1.687–3.970	**< 0.001**	2.136	1.280–3.563	**0.004**
**Verbal harassment by HP (ref: no)**	2.500	1.733–3.606	**< 0.001**	2.017	1.271–3.204	**0.003**
**Sexual harassment by HP (ref: no)**	2.980	1.733–5.126	**< 0.001**	1.475	0.702–3.096	0.305
**Discrimination Experience (ref: no)**	1.807	1.564–2.088	**< 0.001**	1.645	1.404–1.927	**< 0.001**

*LGBT* lesbian, gay, bisexual, and transgender; *ref* reference; *OR* odd ratio; *CI* Confidential interval; ^a^US$1 = RMB7.03; ^b^: only in employed, valid N = 9891; HP: Health Professionals

After controlling for the effects of other variables, seven variables remained significant in the multivariate logistic regression. Divorced/widowed individuals (OR = 1.551, 95%CI: 1.051–2.289), and participants who had religious beliefs (OR = 1.362, 95%CI: 1.176–1.577) had a significantly higher likelihood of utilizing counselling and psychotherapy service. Compared to those who had no psychological distress and/or those who had never experienced discrimination or violence in clinical settings, individuals with previous adverse experience, such as, rejection for treatment by health professionals (OR = 2.136, 95%CI: 1.280–3.563) and verbal harassment from clinicians (OR = 2.017, 95%CI: 1.271–3.204) had an increased likelihood of using the service. Additionally, individuals who were comfortable disclosing their sexual minority identity to others were more likely to accept counselling and psychotherapy service (OR = 2.078, 95%CI: 1.815–2.379).

## Discussion

This is the first study to investigate the prevalence and factors associated with counselling and psychotherapy service utilization among the Chinese sexual minority populations. Similar to previous study findings among a transgender Chinse population, the findings from this study reported similar rates of uptake for counselling and psychotherapy services [19]. There are several issues regarding the counselling and psychotherapy services in Chinese sexual minorities. First, Chinese sexual minorities have limited access to counselling and psychotherapy services due to severe understaffing, which make it difficult to fulfil their mental health needs [22]. Second, a large proportion of Chinese people are unclear about the purpose and nature of counselling and psychotherapy, while others believe that it is only beneficial to white, middle-class individuals and that a western psychological model is not suitable for Chinese populations to work through their psychological distress [23]. Third, Chinese sexual minorities suffer from higher levels of stigma and discrimination than heterosexual people. Chinese society still has a relatively low acceptance of both sexual minority populations and individuals with mental disorders [13].

Sexual minorities usually share greater concerns over discrimination from health services, such as the inequity around the accessibility of HIV prevention and treatment, as well as, a lack of friendliness from staff providing psychological services [1]. Fourth, mental health service costs can be a financial burden to the individual. After the implementation of China’s new National Mental Health Law in 2013 [13], resulted in a number of new treatments now have increased coverage by the National Health Insurance. However, fees for counselling and psychotherapy are still not covered by health insurance, which can make the services unaffordable for many individuals and LGBT individuals can require more counselling and psychotherapy sessions than heterosexual individuals due to the additional level of stigma and discrimination they receive from the general public.

This study found that divorced/widowed individuals were more likely to use counselling and psychotherapy services. This could be partially explained by the uneven distribution of participants in each subtype, only 1.6% of the participants were divorced/widowed and 93.6% of them were single. Compared to single/married sexual minorities, divorced/widowed individuals tended to have more severe psychological distress, poorer psychological adjustment and resilience, which increases the likelihood of service use [24]. In addition, those who have religious beliefs were more likely to utilize counselling and psychotherapy services. Existing evidence has shown there is a positive association between religion and health-promoting behaviours in emerging adults [25]. Researchers, therefore suggest that there should be more emphasis on the role of religion in mental health [26]. This study confirms that religion and mental health continue to remain an important factor in the mental health of LGBT individuals. How this may differ for different religious groups lacks exploration in the Chinese context.

A meta-analysis in 2012 revealed that compared to heterosexual individuals, LGB individuals experienced more victimization, particularly among gay men. The rates for all samples of specific types of victimization ranged from 5% being spat on to 55% experiencing verbal harassment [27]. Our study supports previous evidence that verbal harassment was more commonly experienced than any other form of victimization. In addition, it has been demonstrated that stigma-related victimization persists throughout the life-course for many sexual minorities, and this is associated with poor mental health outcomes (i.e. depressive symptoms) [28, 29]. It is possible that victimization is attributed to an established stressful atmosphere, which consequently generates adverse mental health outcomes [30, 31]. Therefore, people with experiences of victimization are more likely to seek advice from psychological professionals to better appraise and cope with their distress. As there are many different forms of verbal harassment that can result in distress [32], future research should explore how different types of verbal harassment are experienced in the LGBT community.

This study also indicated that perceived discrimination was significantly associated with increased counselling and psychotherapy service utilization. Previous studies showed that the experience of discriminatory events could contribute to poor mental health [6, 27], such as developing Post-Traumatic Stress Disorder (PTSD) [33]. This could be partially explained within the conceptual framework of minority stress in which prejudice, discrimination, and stigma create an unfriendly or even hostile social environment that results in adverse mental health outcomes [30]. However, inconsistent results were also reported. Previous research shows that even though LGBT individuals experience more serious discrimination and report worse mental health outcomes than heterosexuals, discrimination does not significantly account for the disparity or difference in service utilization [5]. These contradictory results could be explained by the difference in research samples (i,e, whether sub-groups were explored or lack of large representative samples) and participants’ sociocultural background. Caution is recommended for future studies that explore the links between discrimination, adverse mental health outcomes, and counselling and psychotherapy service use.

These results also showed that sexual minorities who disclosed their identity to others were more likely to be accepting of and seek out counselling and psychotherapy. Similarly, findings from previous research also indicated that those who disclosed their identity to healthcare professionals were more likely to receive better treatment [34]. In order to meet the specific medical needs of sexual minorities, research further suggests that appropriate training should be provided to health professionals in order to create a friendly clinical setting that facilitates disclosure [34]. Interventions to improve disclosure would be beneficial for individuals in asking for help from professionals and facilitate the ability to achieve self-satisfaction [35]. This study also suggests that disclosure in general, whether family or friends, improves the utilization of counselling and psychotherapy services. Creating an environment in the Chinese context, which facilitates disclosure should be a point for further research [15, 36].

There are several limitations of this study. First, though the national sample size was large, this was a cross-sectional survey. The direct causal associations between potential factors and counselling and psychotherapy service use are tentative. Longitudinal studies are warranted to further identify causal associations. Secondly, all results are based on self-reported data and could not be confirmed with medical records. While most of the questions were designed based on previous findings and expert consultation, they provide weak psychometric evidence, therefore, recall bias and assessment bias may exist. Third, the average age of the current sample was skewed toward a younger age, which may not represent participants from all age groups. It is possible that the utilization of services varies based on the age distribution. Fourth, the majority of the participants were recruited online. Compared with younger generation, it is possible that older generation in China have less access to smart phones and internet. This could lead to the potential exclusion of the older generation. Also, those from a lower socio-economic class might not be able to afford smartphones and internet. Therefore this study was unable to capture those from a lower socio-economic class. In addition, people who did not feel safe to answer online survey or had concerns around privacy of personal data were unlikely to participate in the research. Finally, other relevant variables related to counselling and psychotherapy services utilization, such as, physical comorbidities, family history of mental disorder, and childhood traumatic experiences, were not analysed because the data was unavailable.

## Conclusion

The findings revealed that Chinese sexual minorities had a relatively lower utilization rate for counselling and psychotherapy services. Service providers as well as policy makers should revise policies to address the mental health needs of sexual minority populations by improving the quality of counselling and psychotherapy services such as subsidising the cost of counselling and psychotherapy services. Policy advocacy campaigns are needed to more widely publicised counselling and psychotherapy services within sexual minority communities. It is important to enhance training programs about LGBT specific needs for mental health experts. As well as introduce training programs for healthcare professionals to improve their understanding of sexual minority populations. The development of legal protection for sexual minorities is also highly encouraged to decrease stigma and discrimination that LGBT individuals face.

## Supplementary Information

**Additional file 1.**

## Data Availability

Readers and all interested researchers may contact Runsen Chen (Email address: runsen.chen@psych.ox.ac.uk) for details.

## Abbreviations

LGBT: Lesbian, gay, bisexual and transgender
OR: Odds Ratios
CI: Confidential Interval
