# Supplementary material for: Counselling and psychotherapy service use in Chinese sexual minority populations: a nationwide survey
Source: BMC Psychiatry. 2021 Jan 7;21:11. doi: 10.1186/s12888-020-03010-3 (PMC7791743; doi:10.1186/s12888-020-03010-3)
Supplement: Supplementary file 1 — Additional file 1. [file 12888_2020_3010_MOESM1_ESM.docx]

**Supplementary Table 1. correlation between included variables**

| **Correlations** | | | | | | | | | | | | |
| --- | --- | --- | --- | --- | --- | --- | --- | --- | --- | --- | --- | --- |
|  | 1 | 2 | 3 | 4 | 5 | 6 | 7 | 8 | 9 | 10 | 11 | 12 |
| 1.Age | 1 | -.053^**^ | -.197^**^ | .267^**^ | .329^**^ | -.067^**^ | -.005 | -.040^**^ | .032^**^ | .024^**^ | .010 | -.248^**^ |
| 2.Residency |  | 1 | -.061^**^ | -.241^**^ | -.143^**^ | -.046^**^ | -.014 | -.074^**^ | .012 | .002 | .022^**^ | -.029^**^ |
| 3.Marital Status |  |  | 1 | .009 | -.079^**^ | .014 | .006 | .071^**^ | -.025^**^ | -.011 | -.007 | .089^**^ |
| 4.Education |  |  |  | 1 | .280^**^ | .053^**^ | -.050^**^ | .017^*^ | -.023^**^ | -.015^*^ | -.016^*^ | .024^**^ |
| 5.Yearly Income (RMB) ^a^ |  |  |  |  | 1 | .009 | -.025^*^ | .013 | -.028^**^ | -.012 | -.002 | -.036^**^ |
| 6.Religious |  |  |  |  |  | 1 | .017^*^ | -.009 | -.022^**^ | -.020^**^ | -.027^**^ | .025^**^ |
| 7.Psychological distress |  |  |  |  |  |  | 1 | .046^**^ | .001 | -.023^**^ | -.005 | -.112^**^ |
| 8.Disclosure to others |  |  |  |  |  |  |  | 1 | .008 | .019^**^ | .011 | .114^**^ |
| 9.Refused by HP |  |  |  |  |  |  |  |  | 1 | .161^**^ | .109^**^ | .037^**^ |
| 10.Verbal harassment by HP |  |  |  |  |  |  |  |  |  | 1 | .139^**^ | .044^**^ |
| 11.Sexual harassment by HP |  |  |  |  |  |  |  |  |  |  | 1 | .028^**^ |
| 12.Discrimination Experience |  |  |  |  |  |  |  |  |  |  |  | 1 |
| **. Correlation is significant at the 0.01 level (2-tailed). *. Correlation is significant at the 0.05 level (2-tailed). a: only in employed, valid N=9,891; HP: Health professional; | | | | | | | | | | | | |
